# Mechanistic insights into 5-aminolevulinic acid photodynamic therapy for acne vulgaris: targeting lipogenesis via the OLR1-Wnt/β-catenin pathway

**DOI:** 10.1186/s10020-025-01104-w

**Published:** 2025-02-04

**Authors:** Jia Yan, Linglin Zhang, Qingyu Zeng, Yitao Qian, Ke Li, Xiaojing Liu, Yun Wu, Yu Yan, Haiyan Zhang, Szeman Cheung, Jia Liu, Ronald Sroka, Xiuli Wang, Lei Shi

**Affiliations:** 1 Institute of Photomedicine, Shanghai Skin Disease Hospital, School of Medicine, Tongji University, Shanghai 200092,, China; 2 Laser-Forschungslabor, LIFE Center, University Hospital, Ludwig-Maximilian University, 82152 Planegg, Germany; 3 Department of Dermatology, Huadong Hospital, Fudan University, Shanghai, 200040 P. R. China

## Abstract

Acne vulgaris, a prevalent chronic inflammatory skin disorder, is often characterized by hyperactive sebaceous glands and excessive sebum production, presenting a significant therapeutic challenge. While 5-aminolevulinic acid photodynamic therapy (ALA-PDT) is clinically effective in treating moderate to severe acne, the molecular mechanisms underlying its therapeutic effects remain largely unexplored. In this study, we investigated the impact of ALA-PDT on lipid metabolism in an acne-like mouse model and in immortalized human sebocytes (XL-i-20), focusing on the role of the OLR1-Wnt/β-catenin pathway. We employed transcriptomic analysis, lipid staining, and gene silencing techniques to dissect the molecular interactions induced by ALA-PDT. Our findings revealed that ALA-PDT significantly reduces lipogenesis by upregulating OLR1, which in turn suppresses the SREBP1-FAS axis, thereby decreasing lipid accumulation in sebocytes. Furthermore, activation of the OLR1-Wnt/β-catenin pathway was essential for these lipogenic effects, as silencing OLR1 or activating Wnt/β-catenin signaling reversed lipogenesis inhibition. This study elucidates a novel mechanistic pathway in ALA-PDT-mediated acne treatment, highlighting OLR1 as a promising target for future therapeutic strategies.

## Introduction

Acne vulgaris is a prevalent dermatological condition characterized by chronic inflammation of the pilosebaceous units, affecting almost 85% of adolescents and young adults (Moradi Tuchayi et al. 2015). Its clinical manifestations, including comedones, papules, pustules, nodules and cysts, significantly impact quality of life because of the potential for cosmetic disfigurement and psychological distress (Layton and Ravenscroft 2023). Standard treatments, such as antibiotics and isotretinoin, although effective, are associated with limitations, including antibiotic resistance, hepatotoxicity, teratogenicity, and other adverse effects, underscoring the need for safer and more targeted therapies (Reynolds et al. 2024; Xia et al. 2022).

5-Aminolevulinic acid-based photodynamic therapy (ALA-PDT) has emerged as a promising approach for treating various types of cutaneous diseases, particularly moderate to severe acne (Shi et al. 2021). This therapy involves topical application of ALA, a photosensitizer that selectively accumulates in sebaceous glands and, upon activation by light, generates reactive oxygen species (ROS), including localized cytotoxicity. ALA-PDT is recommended as one of the first-line treatments for moderate to severe acne vulgaris with high efficacy, tolerance and safety (Shi et al. 2021; Zhang et al. 2021a). Compared with systemic isotretinoin, ALA-PDT has demonstrated substantial efficacy in reducing acne lesions, with a favorable safety profile (Zhang et al. 2023). However, despite its therapeutic success, the precise molecular mechanisms by which ALA-PDT mitigates acne symptoms remain insufficiently understood, particularly concerning its effects on sebaceous gland activity and lipid metabolism. ALA can be selectively absorbed by follicular units and sebaceous glands, where it is converted to protoporphyrin IX (Ding et al. 2011). After ALA-PDT, the size of sebaceous glands is reduced, and sebum secretion decreases (Ding et al. 2011; Cao et al. 2021; Wang et al. 2024). The inhibition of excessive sebum secretion is one of the characteristics of ALA-PDT for severe acne vulgaris. However, the mechanism is still unclear.

The oxidized low-density lipoprotein receptor 1 (OLR1) also known as lectin-type oxidized low-density lipoprotein receptor-1 (LOX-1) is a type II membrane glycoprotein receptor that plays an important role in atherosclerosis (Jin and Cong 2019; Sharma et al. 2022). OLR1 is prominently expressed in sebaceous glands, and OLR1 knockdown in SZ95 sebocytes affects the expression of several genes governing cell proliferation and motility (Nagelreiter et al. 2018). The expression of OLR1 in sebaceous glands may be involved in lipogenesis and metabolism. However, there is currently no research indicating a relationship between OLR1 and acne vulgaris. Furthermore, Wnt/β-catenin signaling may be involved in regulating sebaceous differentiation and tissue homeostasis (Augustin 2015; Clayton et al. 2019). It has been reported to regulate de novo lipogenesis and fatty acid monounsaturation (Bagchi et al. 2020). However, whether the OLR1 and Wnt/β‐catenin pathways are linked to decreased sebum secretion after ALA-PDT for acne vulgaris is unknown.

In this study, lipid staining revealed decreased lipid secretion in an acne-like mouse model and XL-i-20 sebocytes after ALA-PDT. Transcriptome microarray analysis revealed that OLR1 was significantly upregulated 24 h after ALA-PDT. Further in vitro experiments indicated that ALA-PDT could significantly upregulate OLR1 expression and inhibit sebum secretion. The above process involves the Wnt/β-catenin pathway. The effects of both oxidized low-density lipoprotein receptor 1 (OLR1) and the Wnt/β-catenin signaling pathway, which are implicated in lipid metabolism, on sebocytes have not yet been explored. This study aimed to elucidate the role of the OLR1-Wnt/β-catenin axis in mediating ALA-PDT-induced lipogenesis inhibition, providing novel insights into its therapeutic mechanism in acne vulgaris.

## Methods

### Reagents

ALA hydrochloride powder was obtained from Fudan Zhangjiang Biopharmaceutical Co., Ltd. (Shanghai, China). The β-catenin inhibitor XAV-939 and the specific agonist KY19382 were purchased from Med Chem Express (NJ, USA). OLR1 siRNA was purchased from Hanbio Biotechnology Co., Ltd. The following reagents were used: anti-OLR1 antibody (ab60178, Abcam), anti-SREBP-1 antibody (AF6283, Affinity), anti-FAS antibody (ab128870, Abcam), anti-beta-catenin antibody (8480 S, CST), and anti-GAPDH antibody (AF7021, Affinity). BODIPY 493/503 was obtained from Thermo Fisher Scientific (Germany).

### Acne-like mouse model construction

The skin commensal Propionibacterium acnes (P. acnes), recently renamed Cutibacterium acnes (C. acnes), are used to intracutaneous injected to the back of hairless mice to establish a robust acne-like mouse model (Ladopoulos et al. 2024). The C. acnes (ATCC^®^ strain 6919™) obtained from the American Type Culture Collection (Manassas, VA) was cultured in BHI liquid media at 37 °C under anaerobic conditions created by AnaeroPack (Mitsubishi Gas Chemical Company, Tokyo, Japan). C. acnes in the logarithmic growth phase were collected after centrifugation at 5000 × g for 10 min and suspended in PBS. Six- to eight-week-old SKH-1 female mice obtained from the Public Health Clinical Center were used for the acne-like mouse model. The content and composition of mouse sebum differ from human sebum, and the former has significantly lower triglycerides and squalene. In order to promote the persistence of intradermally injected C. acnes to develop the acne murine model, Kolar, Stacey L et al. applied synthetic human sebum immediately following injection and reapplied daily (Kolar et al. 2019). Adhering to the methodology outlined by O’Neill, Alan M et al., the squalene, a principal component of human sebum and found in higher concentrations in sebum of subjects with acne, was topically applied to the local region prior to and following injection (O’Neill et al. 2022). Specifically, one day before injection and every 24 h thereafter, 100 µl of squalene (Aladdin, S109119) was applied to the right back. In addition, 20 µl of living C. acnes bacterial mixture (1 × 10^7^ CFU) was intradermally injected into the right back under anesthesia with isoflurane, while only an equal volume of PBS was injected into another side as control. Three days later, the lesions were harvested, and histomorphology examination was performed. The study protocol for the animals was approved by the Ethics Committee of Shanghai Skin Disease Hospital.

### Cell culture

The H-tert gene was subsequently transferred into primary human sebocytes to construct the immortalized cell line XL-i-20 (patent number: ZL 202011577037.2) (Liu et al. 2024). The cells were cultivated in DMEM supplemented with 10% fetal bovine serum (Gibco, Carlsbad, CA, USA), 100 U/ml penicillin and 100 µg/ml streptomycin at 37 °C with 5% CO2.

### ALA-PDT in vitro and in vivo

For in vitro ALA-PDT, XL-i-20 sebocytes were incubated with ALA (0.5 mM) in serum-free DMEM for 4 h in the dark and then washed with Hank’s balanced salt solution (HBSS) twice. The samples were irradiated with 5 J/cm^2^ and 10 J/cm^2^ red light (635 ± 5 nm) and then cultured for 6–24 h for further experiments.

For in vivo ALA-PDT, Acne-like mouse models were established on both sides. Three days after injection, 5% ALA cream was topically applied to the lesion area on one side of the back and incubated in the dark for 3 h. The residual cream was removed, and the lesion area was irradiated with red light (635 ± 5 nm) at a power density of 80 mW/cm^2^ and an energy density of 20 J/cm^2^ when the other skin was protected from light. The untreated half back was used as a control. Demoscopic pictures were taken daily, and the volumes of the lesions were recorded. Histomorphology examination was performed at 24 h, 1 week, 2 weeks, and 4 weeks after treatment.

### Transcriptome microarray analysis

Patients with severe acne diagnosed by two board-certified dermatologists were included. The patients were subsequently screened according to the inclusion and exclusion criteria after providing informed consent. Finally, 3 patients received ALA-PDT, and two nodule lesions were punched before and 24 h after ALA-PDT for transcriptome microarray analysis. The use of human samples was approved by the Ethics Committee on Human Subject Research at Shanghai Skin Disease Hospital. Total RNA was extracted from the lesion samples via TRIzol reagent (Invitrogen) and quantified via a NanoDrop (Invitrogen). Microarray analysis was performed via the Affymetrix GeneChip^®^ Human Transcriptome Array 2.0 (Affymetrix). The data were analyzed via the Transcriptome Analysis Console (TAC, Affymetrix). Genes with *P* values < 0.05 and absolute values of fold change > 2 (|log2FC| > 1) were considered differentially expressed genes (DEGs) in the comparison between before and 24 h after ALA-PDT. The downregulated noncoding RNAs were functionally annotated via the R package “clusterProfiler” for Gene Ontology (GO) analysis (Wu et al. 2021). A statistically significant threshold was set at an adjusted *p* < 0.05. The upregulated DEGs were intersected with 1110 lipid metabolism-related genes (LMRGs) from the MSigDB database to identify overlapping genes (Castanza et al. 2023).

### Evaluation of protoporphyrin IX (PpIX) and ROS generation

XL-i-20 sebocytes were incubated with ALA (0.5 mM) in serum-free DMEM for 0–24 h in the dark and then washed with HBSS. After the cells were cocultured with 1 mL of dimethyl sulfoxide (DMSO) for 30 min, the solution was detected with a fluorescence spectrophotometer at an excitation wavelength of 405 nm. The normalized fluorescence intensity at approximately 630 nm was measured to evaluate the concentration of PpIX.

The XL-i-20 sebocytes were divided into four groups: control, ALA only, red light only and ALA-PDT, and treated with ALA (0.5 mM, 4 h) and/or red light (635 ± 5 nm, 5 J/cm^2^). The cells in each group were incubated with the ROS fluorescent probe DCFH-DA for 30 min, followed by washing twice with HBSS. The fluorescence images were observed via a fluorescence microscope (excitation: 502 nm, emission: 530 nm).

### Cell viability assay

XL-i-20 sebocytes were incubated with ALA (0.5 mM) for 0–4 h and irradiated with red light (635 ± 5 nm) for 0, 2, 5, or 10 J/cm^2^. Twenty-four hours after treatment, cell viability was measured with a Cell Counting Kit-8 (CCK-8) to evaluate the cytotoxicity of ALA-PDT to sebocytes. The XL-i-20 sebocytes were divided into four groups: control, ALA only, red light only and ALA-PDT. Three hours after treatment, the cells were stained with the live cell indicator calcein-AM and the dead cell indicator PI for 30 min. Fluorescence images were acquired via a fluorescence microscope (excitation: 490 nm, emission: 515 nm; excitation: 535 nm, emission: 615 nm).

### Intracellular lipid staining and quantification

To quantify the intracellular lipid content, droplets of XL-i-20 sebocytes were labeled with Nile red fluorescent stain and BODIPY 493/503 (4 µM in PBS). For Nile red staining, XL-i-20 sebocytes were incubated with 10 µg/ml Nile red dissolved in DMSO for 10 min and then observed under a fluorescence microscope. For BODIPY 493/503 staining, the cell slides were incubated with 4 µM BODIPY 493/503 dissolved in PBS for 30 min after fixation. The nucleus were identified by DAPI staining and then observed under a confocal microscope (Nikon, Japan). The number of lipid droplets was determined via ImageJ, and the average number of lipid droplets per cell was calculated to evaluate the degree of lipogenesis.

### Quantitative real-time PCR (RT‒PCR)

The relative expression level of OLR1 was analyzed via RT‒PCR. The skin tissues were harvested and ground in liquid nitrogen. Total RNA was extracted from skin tissue and XL-i-20 sebocytes via the TRIzol reagent (Invitrogen, USA) and then reverse transcribed to cDNA via the Maxima H Minus First Strand cDNA Synthesis Kit with dsDNase (Thermo Fisher Scientific).

### Histomorphology analysis and immunohistochemistry (IHC)

The model mice were humanely sacrificed before and 24 h, 1 w, 2 w, 4 w after ALA-PDT. The tissues were stored in formalin, and 5-µm sections were dewaxed, followed by hematoxylin and eosin (H&E) staining. The slides were placed at 60 °C for 30 min and deparaffinized, followed by rehydration in xylene and ethanol. After blocking with 10% BSA, the samples were incubated with an anti-OLR1 antibody (ab60178, Abcam) overnight at 4 °C.

### Western blot analysis

Total protein was extracted from acne-like mouse model tissues and XL-i-20 sebocytes with RIPA buffer. Then, the protein samples were separated on 10% and 7.5% SDS‒polyacrylamide gels and transferred to PVDF membranes. After being blocked in 5% nonfat milk for 1 h, the membranes were incubated with primary antibodies overnight at 4 °C. Following 5 washes with TBST, the membranes were incubated with an HRP-conjugated goat anti-rabbit secondary antibody (Cell Signaling Technology) for 1 h. The bands were visualized via enhanced chemiluminescence.

### Cell transfection

The XL-i-20 sebocytes were seeded into 6-well plates and allowed to grow to 40–50% confluence before being transiently transfected with OLR1 siRNA (Hanbio Biotechnology Co. Ltd., Shanghai, PR China) and RNAFit. Nonspecific siRNA was transfected as a negative control (NC-siRNA). After transfection for 48 h, the silencing effect of OLR1 was confirmed through RT‒PCR and Western blot.

### Statistical analysis

The data were based on three independent experiments, interpreted as the means ± SDs and were analyzed via Prism software (GraphPad Software, Inc.). Comparisons between two groups were conducted via an unpaired, two-tailed Student’s t test. One-way or two-way ANOVA was used for multiple comparisons, followed by the post hoc Bonferroni correction. *P* < 0.05 was considered statistically significant.

## Results

### ALA-PDT suppresses lipogenesis in an acne-like mouse model

**Fig. 1:**
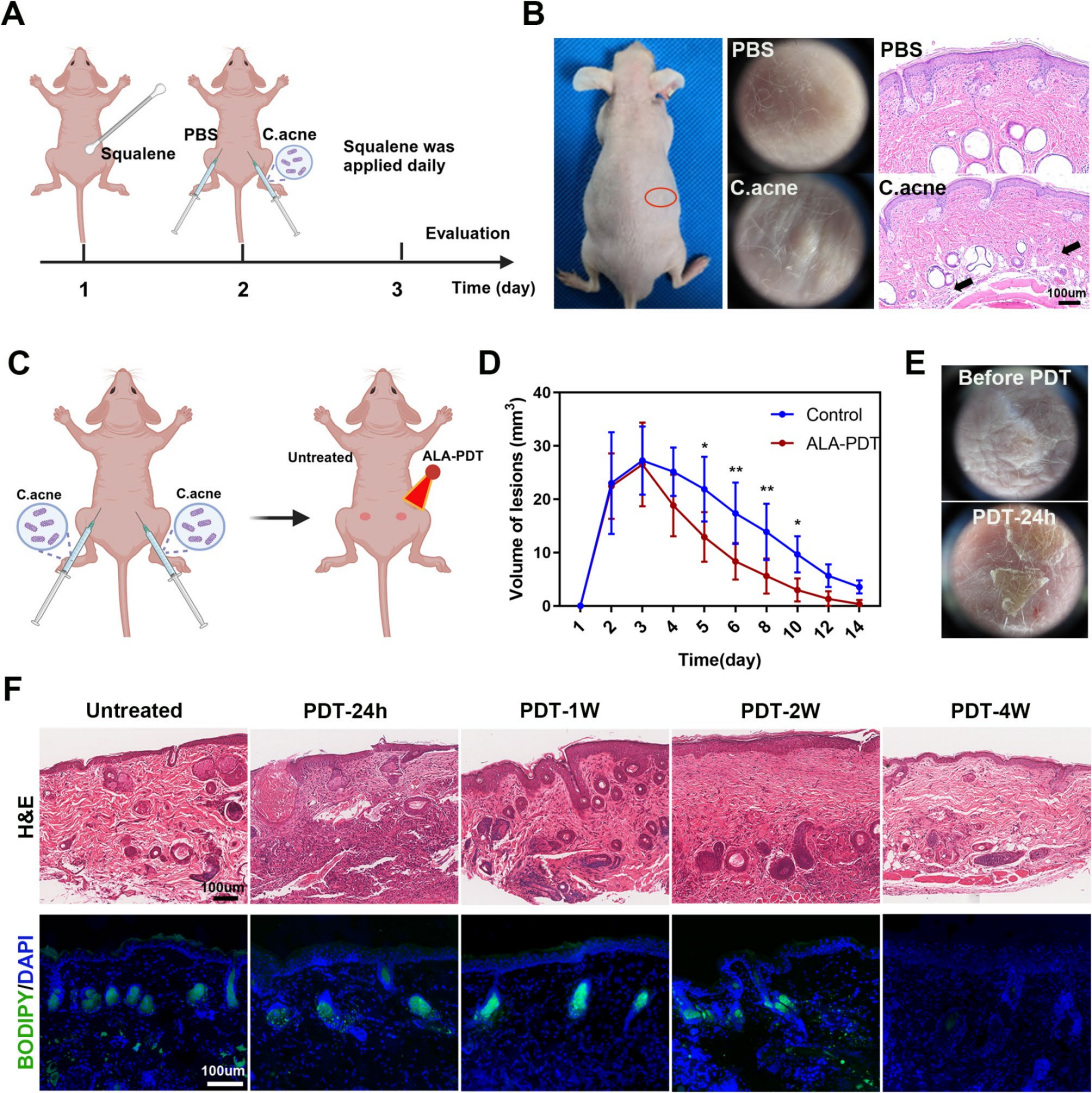
The therapeutic effect of ALA-PDT in an acne-like mouse model. (**A**) Schematic of the acne-like mouse model. Intradermal injection of 10^7^ CFU of living C. acnes suspended in PBS. PBS injection was used as a control. (**B**) Dermoscopic and H&E staining images of the acne-like mouse model. (**C-D**) Changes in the volume of lesions on the back of the acne-like mouse model after ALA-PDT. (**E**) Representative dermoscopic images of lesions before and 24 h after ALA-PDT. (**F**) H&E staining and BODIPY staining of the tissues of the acne-like mouse model before and 24 h, 1 w, 2 w, 4 w after ALA-PDT. *P < 0.05, **P < 0.01

An acne-like mouse model was generated with C. acnes and squalene in this study (Fig. 1A). After injection, a papule was observed on the dorsal region of the mouse, which was examined via digital photography and dermatoscopy. H&E staining revealed inflammatory cell infiltration surrounding the pilosebaceous unit (black arrow in Fig. 1B), which is similar to the inflammatory papules of acne vulgaris. To evaluate the effect of ALA-PDT in vivo, an acne-like mouse model was subjected to ALA-PDT. Specifically, 10^7^ CFU of living C. acnes in PBS were intradermally injected into both backs of each mouse after the application of squalene, and ALA-PDT was conducted on the lesion of one side, while the other side was used as an untreated control (Fig. 1C). The volume changes of the lesions are shown in Fig. 1D, and the lesions on the side treated with ALA-PDT subsided significantly faster than those on the untreated side did, although they could also resolve spontaneously within approximately 2 weeks. As shown in Fig. 1E, a thin, pale-yellow crust was observed on the surface of the lesions 24 h post-treatment, a phenomenon that is similar to clinical observations. H&E staining before and after ALA-PDT revealed significant inflammation after treatment, which gradually subsided with time, which is consistent with previous studies (Zhang et al., 2021b) (Fig. 1F). BODIPY staining of frozen sections revealed a reduction in sebaceous glands and lipid droplets after treatment. These results suggested that ALA-PDT exerts antical effects in vivo, promoting the regression of lesions and reducing sebaceous glands in an acne-like mouse model.

**Fig. 2:**
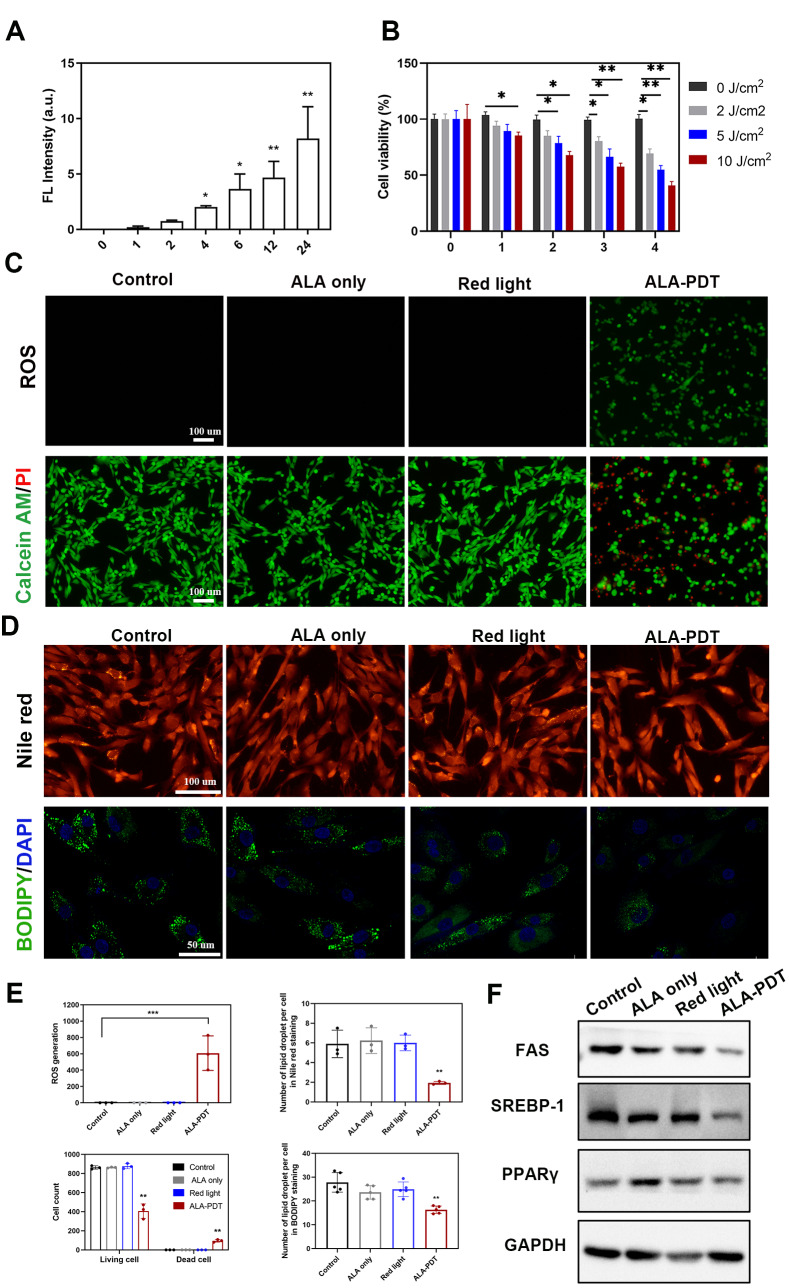
ALA-PDT inhibits lipid accumulation in XL-i-20 sebocytes. (**A**) Fluorescence intensity of XL-i-20 sebocytes after incubated with 0.5 mM ALA for 0–24 h detected via a fluorescence spectrophotometer. (**B**) Viability of XL-i-20 sebocytes incubated with 0.5 mM ALA for different durations and illuminated with red light at different doses. (**C**) ROS production and live/dead cell staining of XL-i-20 sebocytes were observed via fluorescence microscopy. (**D**) Nile red staining and BODIPY staining of XL-i-20 sebocytes 24 h after ALA-PDT. (**E**) ROS semiquantitative analysis and counting of living/dead cells in 2 C. Semiquantitative analysis of lipid droplets per cell in 2D. (**F**) Western blot of FAS, SREBP1 and PPARγ proteins in XL-i-20 sebocytes 24 h after ALA-PDT. *P < 0.05, **P < 0.01

### ALA-PDT inhibits lipid accumulation in XL-i-20 sebocytes

To explore the effect of ALA-PDT on XL-i-20 sebocytes, the fluorescence of PpIX after incubation with ALA and the inhibitory effect of ALA-PDT were examined. After incubation with 0.5 mM ALA for 0–24 h, the centuriation of PpIX was detected via a fluorescence spectrophotometer at an excitation wavelength of 405 nm. As shown in Fig. 2A, the concentration of PpIX gradually increased with increasing incubation time, and there was a significant difference in intensity from 4 h. Considering the damage to cells caused by starvation culture during the incubation of ALA, a shorter incubation time was adopted for in vitro experiments. The cell viability was subsequently detected after treatment with different doses of ALA-PDT (Fig. 2B). The results revealed that cell viability decreased with increasing incubation time and increasing light dose. Therefore, an in vitro parameter of 4 h incubation time and a light dose of 5 J/cm^2^ was chosen for subsequent experiments for that these conditions could achieve approximately half the kill. The fluorescence probe DCFH-DA was used to detect ROS generation (Fig. 2C, E), and ROS were detected in only the ALA-PDT group. In addition, the dyes Calcin-AM and PI were used to label living and dead cells, respectively (Fig. 2C,E). ALA alone and red light at the experimental doses caused almost no cell death, whereas ALA-PDT caused partial cell death, as indicated by the red fluorescence in the figures. To substantiate the inhibitory effect on lipid accumulation, Nile red staining and BODIPY staining were used to label the lipid droplets, and the number of lipid droplets was counted with ImageJ (Fig. 2D-E). The number of lipid droplets per cell in the ALA-PDT group was significantly reduced. The transcription factor SREBP1 and peroxisome proliferator activated receptor gamma (PPARγ), which critically affect diverse downstream genes involved in lipid biosynthesis, play important roles in sebum overproduction in acne vulgaris (Li et al. 2023; Yang et al. 2021). It has been reported that some antiacne drugs or therapies can regulate the expression of SREBP1, PPARγ and its downstream gene fatty acid synthase (FAS) to suppress lipogenesis (Shin et al. 2022; Lu et al. 2019). Owing to the important role of SREBP-1, PPARγ and FAS in lipid homeostasis, the protein levels 24 h after treatment were detected via western blot (Fig. 2F), and the results revealed that ALA-PDT decreased the expression of FAS, SREBP-1, and PPARγ. These results suggested that XL-i-20 sebocytes could produce PpIX after ALA incubation and generate ROS after red-light illumination to suppress cell viability. ALA-PDT could reduce lipid accumulation in XL-i-20 sebocytes and downregulate the expression of the lipid synthesis-related proteins SREBP-1, PPARγ and FAS. The findings are in line with previous observations in primary sebocytes and SZ95 cells (Yang et al. 2021; Tuo et al. 2017).

### ALA-PDT induces the upregulation of OLR1 in XL-i-20 sebocytes and the acne-like mouse model

**Fig. 3:**
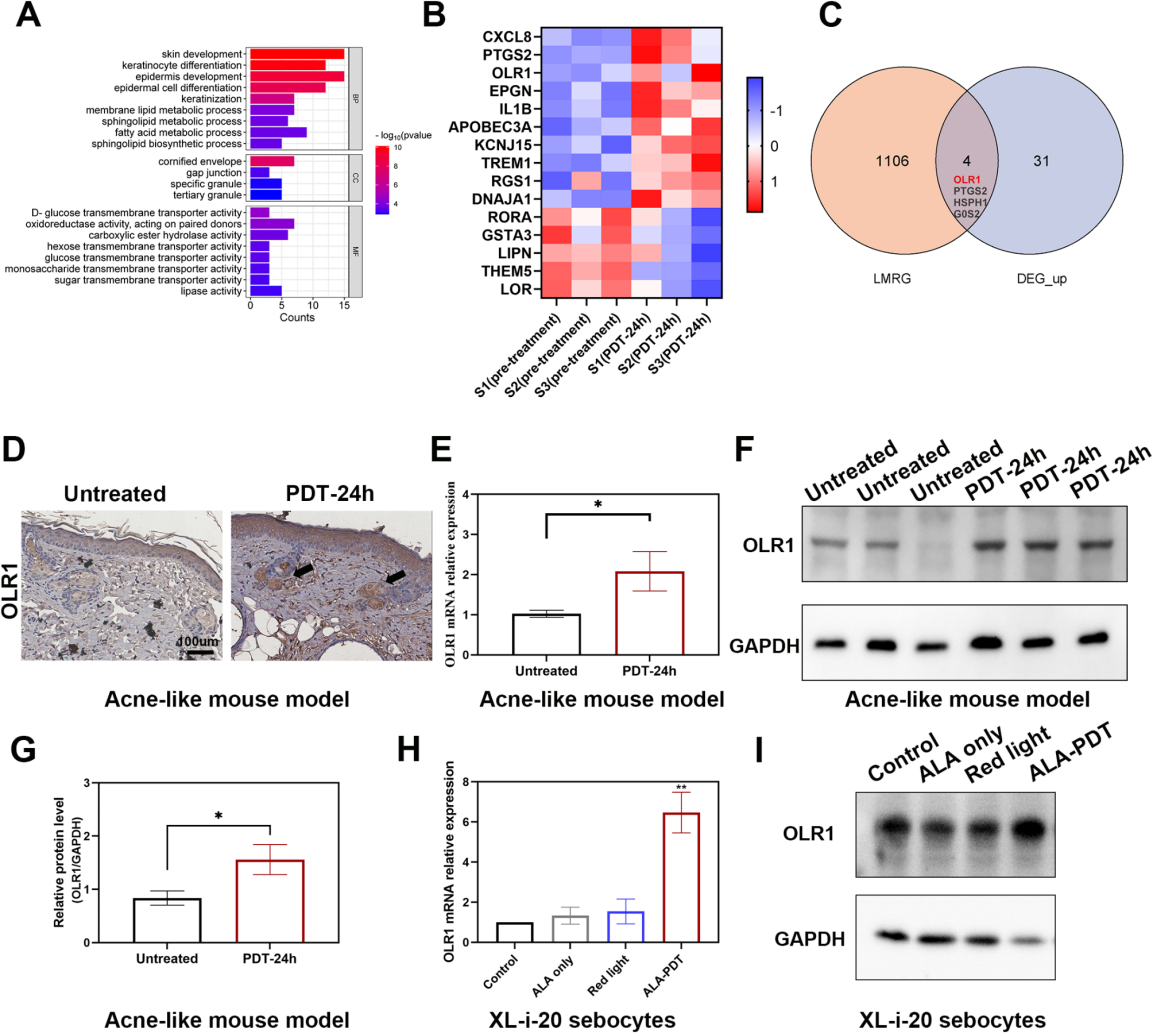
ALA-PDT induces the upregulation of OLR1 in XL-i-20 sebocytes and the acne-like mouse model. (**A**) GO analysis of downregulated DEGs. (**B**) Heatmap of DEGs of interest in acne lesions before and 24 h after ALA-PDT (*n* = 3). (**C**) The intersection of lipid metabolism-related genes (LMRGs) and upregulated DEGs. (**D**) IHC staining for OLR1 in the acne-like mouse model in the untreated group and at 24 h after ALA-PDT. (**E**) Relative mRNA level of OLR1 in the acne-like mouse model, both untreated and 24 h after ALA-PDT. (**F-G**) Western blot of relative OLR1 protein expression in the acne-like mouse model 24 h after ALA-PDT. (**H-I**) Relative mRNA and protein levels of OLR1 in XL-i-20 sebocytes in the control, ALA-only, red light-only and ALA-PDT groups. GAPDH was used as a control. *P < 0.05, **P < 0.01, *n* = 3

To explore the mechanism of ALA-PDT for the treatment of acne vulgaris, lesions from 3 patients with acne vulgaris were isolated before and 24 h after ALA-PDT for transcriptome microarray analysis. The GO analysis of the downregulated differentially expressed coding RNAs revealed several significantly enriched GO terms related to lipid metabolism, including sphingolipid biosynthetic process, fatty acid metabolic process, sphingolipid metabolic process and lipase activity (Fig. 3A). The heatmap shows the DEGs of interest, where several genes, including CXCL8, PTGS2 and OLR1, were significantly upregulated in the PDT-24 h group (Fig. 3B). Our previous studies revealed the important role of CXCL8 and PTGS2 in the regulation of inflammation (Zhang et al., 2021b; Liu et al. 2023). It is hypothesized that the modulation of lipid metabolism may be the key role of ALA-PDT in preventing acne. Then, four overlapping genes, OLR1, G0S2, HSPH1, and PTGS2, were identified by intersecting lipid metabolism-related genes with upregulated DEGs (Fig. 3C). We speculated that OLR1 was most likely the key gene in acne treatment by ALA-PDT for which has been reported to clear oxidized lipids, whereas the other three genes may promote the secretion of sebum.

To validate the transcriptome sequencing results, the expression of OLR1 was determined through immunohistochemical (IHC) staining, quantitative real-time PCR (RT‒PCR) and western blot (WB). The acne-like model mice were treated with ALA-PDT, and lesions were isolated 24 h later. IHC staining revealed higher levels of OLR1 expression at the sebaceous gland site than in the untreated group (Fig. 3D), and both the mRNA and protein levels of OLR1 were elevated by ALA-PDT in the acne-like mouse model (Fig. 3E-G). Consistent with the in vivo results, the mRNA and protein levels of OLR1 were also increased in XL-i-20 sebocytes upon ALA-PDT treatment (Fig. 3H-I). Taken together, these results revealed that ALA-PDT specifically upregulated OLR1 both in vitro and in vivo.

### ALA-PDT suppresses lipogenesis in XL-i-20 sebocytes via the Wnt/β-catenin pathway

**Fig. 4:**
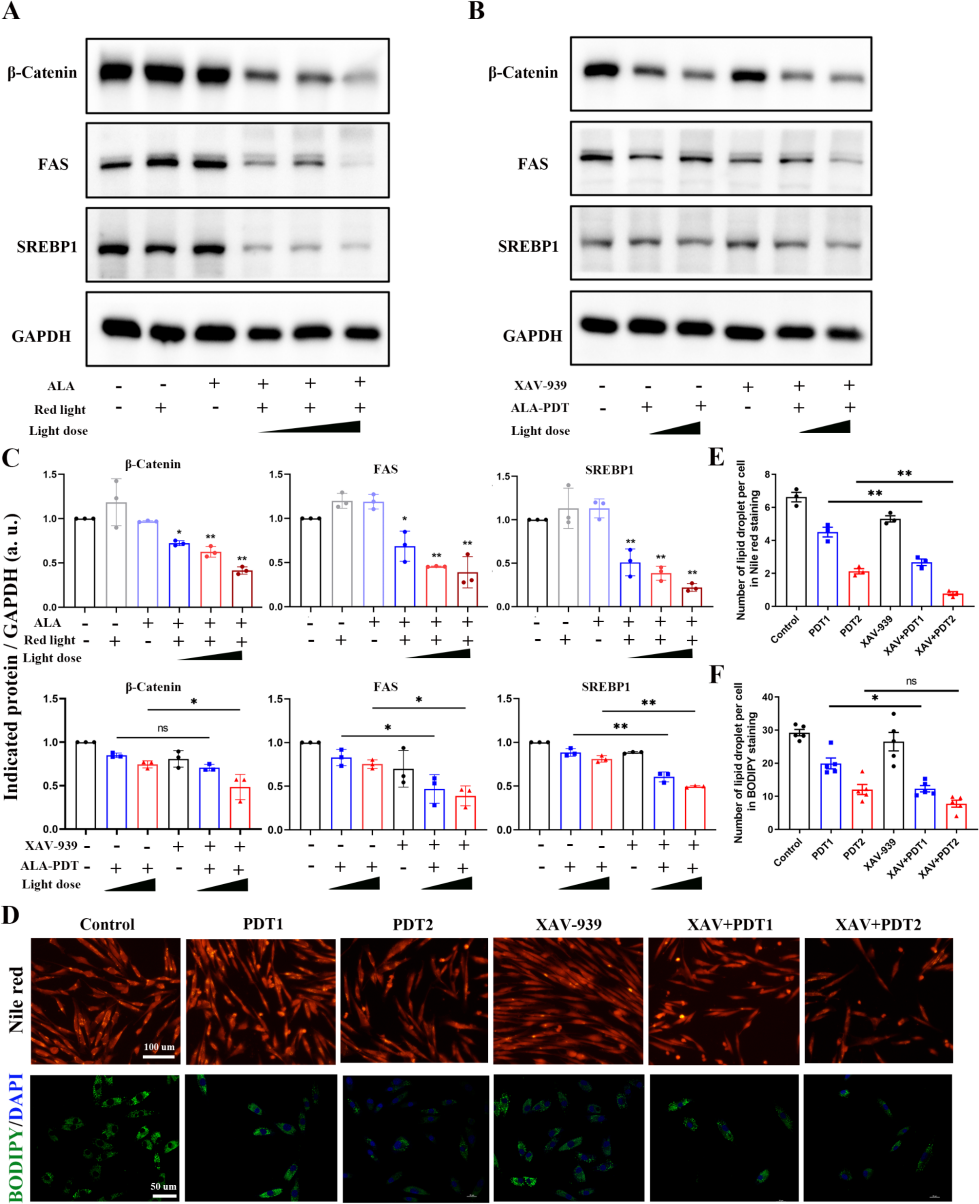
ALA-PDT suppresses lipogenesis in XL-i-20 sebocytes via the Wnt/β-catenin pathway. (**A**) Western blot of relative β-catenin, FAS, and SREBP1 protein expression 24 h after ALA-PDT with different light doses. GAPDH was used as a control. (**B**) Representative protein expression level of β-catenin, FAS, and SREBP1 with or without XAV-939 pre-treatment and different doses of ALA-PDT. GAPDH was used as a control. (**C**) The relative expression levels of β-catenin, FAS, and SREBP1 were semi-quantified. Similar results were observed in three independent experiments. (**D**) Nile red staining and BODIPY 493/503 staining of XL-i-20 sebocytes 24 h after ALA-PDT with or without XAV-939 pre-treatment. (**E-F**) Semiquantitative analysis of lipid droplets per cell in 4D. XAV-939 is an inhibitor of Wnt/β-catenin, and PDT1 and PDT2 means ALA-PDT with light doses of 5 J/cm^2^ and 10 J/cm^2^, respectively. XAV + PDT1 and XAV + PDT2 indicate the combination of XVA-939 and different doses of ALA-PDT. * indicates *p* < 0.05, ** indicates *p* < 0.01, *n* = 3

The Wnt/β-catenin pathway is implicated in the fate determination of stem cells residing in the hair follicle junction zone (JZ) and supporting sebaceous gland homeostasis (Clayton et al. 2019; Shang et al. 2022). In addition, it has been reported that in adipocytes, the Wnt/β-catenin pathway regulates de novo lipogenesis and mediates lipid metabolism through the transcriptional regulation of SREBP1 (Bagchi et al. 2020). To further study the mechanism by which ALA-PDT suppresses lipogenesis, we detected the expression level of β-catenin after ALA-PDT. The western blot results shown in Fig. 4A revealed inhibited expression of β-catenin, FAS, and SREBP1 in XL-i-20 sebocytes after ALA-PDT in a light dose-dependent manner (Fig. 4A, C). It is speculated that ALA-PDT suppresses lipogenesis via the Wnt/β-catenin pathway, and to validate this hypothesis, an inhibitor of the Wnt/β-catenin pathway XAV-939 was used. The XL-i-20 sebocytes were pre-treated with 10 µM XAV-939 for 2 h before ALA-PDT. Compared with the untreated groups, XAV-939 inhibited the expression of β-catenin and further downregulated the FAS and SREBP1 induced by ALA-PDT (Fig. 4B, C). The lipid droplet staining of XL-i-20 sebocytes shown in Fig. 4D revealed that ALA-PDT decreased the number of lipid droplets in a light dose-dependent manner and XAV-939 synergistically inhibited the synthesis of lipid droplets (Fig. 4D-F). Together, these studies provide evidence that ALA-PDT suppresses lipogenesis in XL-i-20 sebocytes via the Wnt/β-catenin pathway.

### Activation of Wnt/β-catenin prevents the reduction in lipogenesis in ALA-PDT-treated XL-i-20 sebocytes

**Fig. 5:**
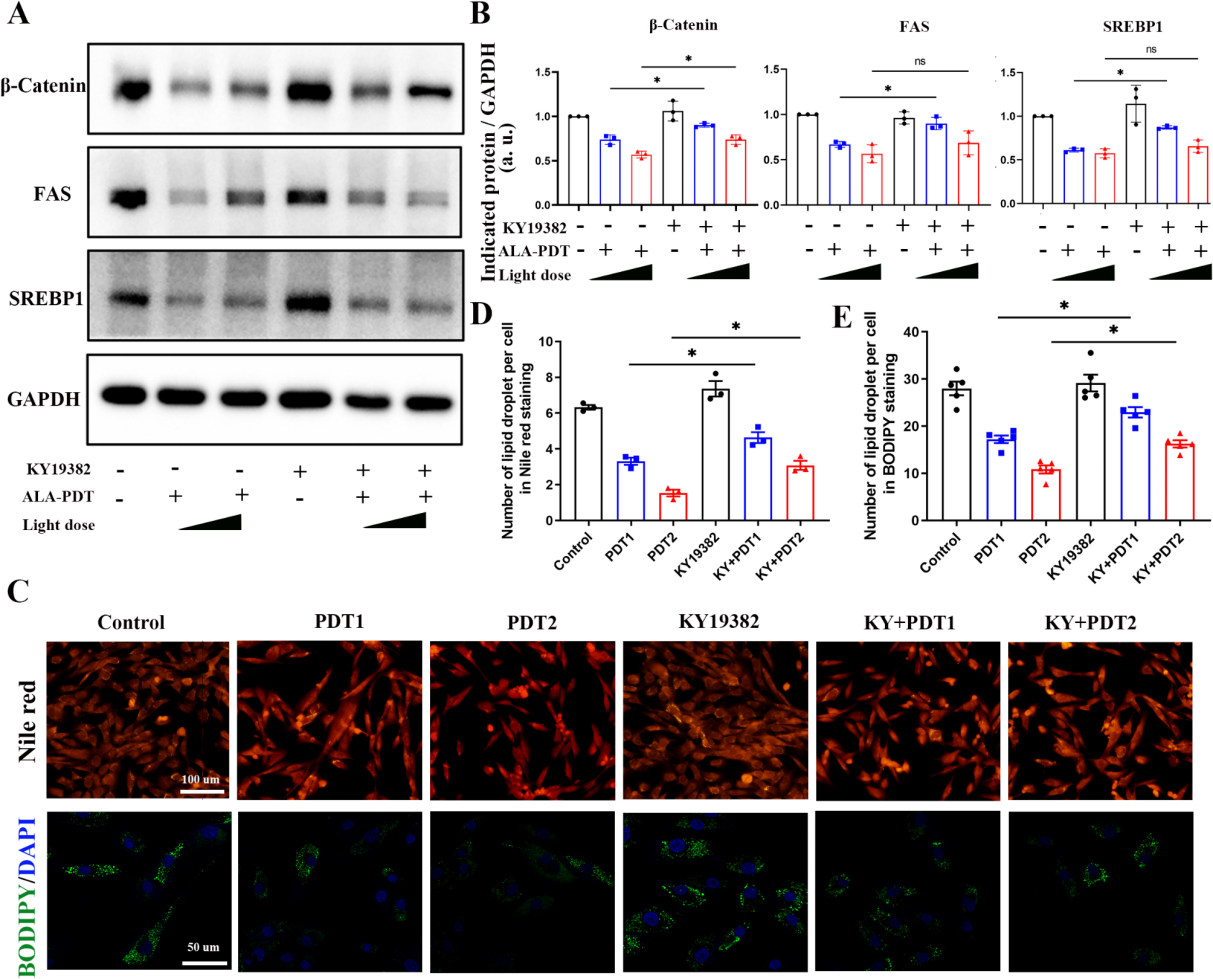
ALA-PDT-mediated sebosuppressive effects on XL-i-20 sebocytes were reversed by Wnt/β-catenin pathway activation. (**A**) Relative protein expression of β-catenin, FAS, and SREBP1 with or without KY19382 pre-treatment and different doses of ALA-PDT. GAPDH was used as a control. (**B**) The relative expression levels of β-catenin, FAS, and SREBP1 were semi-quantitatively analyzed. Similar results were observed in three independent experiments. (**C**) Nile red staining and BODIPY 493/503 staining of XL-i-20 sebocytes 24 h after ALA-PDT with or without KY19382 pre-treatment. (**D-E**) Semiquantitative analysis of lipid droplets per cell in 5 C. KY19382 is an activator of Wnt/β-catenin, and PDT1 and PDT2 means ALA-PDT with light doses of 5 J/cm^2^ and 10 J/cm^2^, respectively. KY + PDT1 and KY + PDT2 indicate the combination of KY19382 and different doses of ALA-PDT. * indicates *p* < 0.05, ** indicates *p* < 0.01, *n* = 3

The Wnt/β-catenin activator KY19382 was used to evaluate the lipid content and the expression of lipid-related proteins in XL-i-20 sebocytes to determine whether the Wnt/β-catenin signaling pathway is required for the sebosuppressive effects of ALA-PDT (Ryu et al. 2021). The western blot results in Fig. 5A showed elevated expression levels of β-catenin treated with 1 µM KY19382 for 4 h. In addition, the results demonstrated that ALA-PDT downregulated the expression of β-catenin, FAS, and SREBP1, but this effect was reversed by the Wnt/β-catenin activator KY19382 (Fig. 5A-B). The intracellular lipid levels in XL-i-20 sebocytes were subsequently identified via Nile red staining and BODIPY 493/503 staining. To a certain extent, ALA-PDT suppressed lipogenesis in XL-i-20 sebocytes in a light dose-dependent manner. However, these effects were diminished by KY19382 (Fig. 5C-E). These findings suggest that Wnt/β-catenin is involved in ALA-PDT-mediated sebosuppressive effects and the activation of Wnt/β-catenin could reverse this inhibitory effect.

### OLR1 knockdown antagonizes the suppression of lipogenesis and inhibition of the Wnt/β-catenin pathway induced by ALA-PDT in XL-i-20 sebocytes

**Fig. 6:**
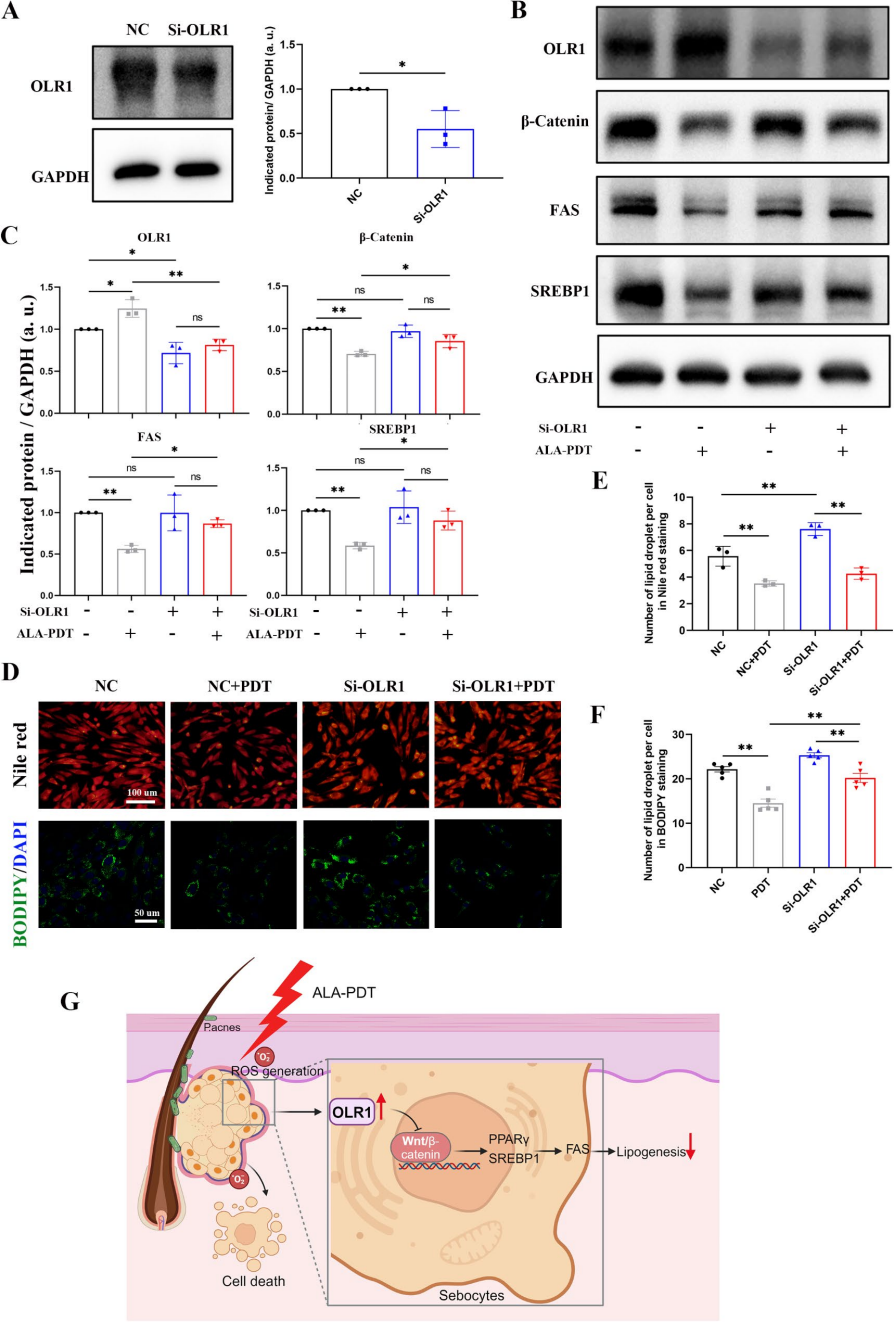
ALA-PDT downregulates the Wnt/β-catenin pathway to inhibit lipogenesis in an OLR1-dependent manner. (**A**) XL-i-20 sebocytes were transfected with NC-siRNA or OLR1-siRNA for 48 h, and western blot was performed to evaluate the expression levels of OLR1. GAPDH was used as a control. Similar results were observed in three independent experiments. (**B**) Relative protein expression of OLR1, β-catenin, FAS, and SREBP1 24 h after ALA-PDT in the NC-siRNA- or OLR1-siRNA-transfected XL-i-20 sebocytes. GAPDH was used as a control. (**C**) The relative expression levels of OLR1, β-catenin, FAS, and SREBP1 were semi-quantified. Similar results were observed in three independent experiments. (**D**) Nile red staining and BODIPY 493/503 staining of the NC-siRNA- or OLR1-siRNA-transfected XL-i-20 sebocytes 24 h after ALA-PDT. (**E-F**) Semiquantitative analysis of lipid droplets per cell in 6D. (**G**) ALA-PDT upregulated OLR1 to inhibit the Wnt/β-catenin pathway and downregulated FAS and SREBP1 to suppress lipogenesis. NC indicates the negative control, and Si-OLR1 indicates OLR1-siRNA. NC + PDT and Si-OLR1 + PDT indicate that NC-siRNA- or OLR1-siRNA-transfected XL-i-20 sebocytes were treated with ALA-PDT. * indicates *p* < 0.05, ** indicates *p* < 0.01, *n* = 3

Previous studies have reported that there is a regulatory relationship between OLR1 and the Wnt/β-catenin pathway to inhibit the accumulation of lipids (Zhang et al. 2015, c). Thus, a small interfering RNA (siRNA) was used to knock down OLR1 in XL-i-20 sebocytes to evaluate whether OLR1 could affect the inhibitory effects of ALA-PDT on lipogenesis and the Wnt/β-catenin pathway. Experiments were conducted within two passages post-transfection. Compared with negative control (NC)-siRNA, si-OLR1 effectively downregulated the expression of OLR1 in sebocytes (Fig. 6A). ALA-PDT upregulated the expression of OLR1 and downregulated the Wnt/β-catenin pathway to inhibit the expression of the lipid synthesis-related proteins FAS and SREBP1 in NC-siRNA-transfected XL-i-20 sebocytes, which was consistent with previous findings. However, these inhibitory effects were abolished in OLR1-knockdown XL-i-20 sebocytes (Fig. 6B-C). Specifically, ALA-PDT only slightly decreased the expression levels of β-catenin and lipid synthesis-related proteins in OLR1-knockdown XL-i-20 sebocytes with no statistical difference. As shown in Fig. 6D-F, compared with the NC group, the knockdown of OLR1 increased the number of lipid droplets in XL-i-20 sebocytes. While ALA-PDT was conducted, the number of lipid droplets in Si-OLR1 sebocytes was significantly greater than that in the NC group, demonstrating that the knockdown of OLR1 counteracts the effects of ALA-PDT. The decreased rate of lipid droplets was higher in NC groups than that in Si-OLR1 groups (0.34 vs. 0.20) under the BODIPY staining, but similar under the Nile red staining (0.37 vs. 0.38). However, ALA-PDT could decrease the number of lipid droplets to some extent in the Si-OLR1 sebocytes. This phenomenon may indicate that there is a complex regulatory network involved in the mechanism of ALA-PDT inhibiting lipid synthesis. In addition, the limited efficiency of OLR1 knockdown using siRNA transient transfection in XL-i-20 cells may have some impact on the outcomes. Overall, these results suggested that ALA-PDT might suppress lipogenesis through the OLR1-Wnt/β-catenin pathway.

## Discussion

ALA-PDT has gained recognition as an effective therapeutic strategy for moderate to severe acne vulgaris, particularly because of its ability to control inflammation and reduce sebum production. However, despite its clinical success, the precise mechanism underlying the effects of ALA-PDT remain incompletely understood. Previous studies have demonstrated that ALA-PDT can reduce the size of sebaceous glands, suppress cell growth in sebocytes, and inhibit lipid secretion through various signaling pathways, such as the mTOR, AMPK, and SREBP-1 pathways (Yang et al. 2021; Tuo et al. 2017; Liu et al. 2018). In our study, we observed rapid resolution of acneiform lesions, with histopathological findings indicating a transient exacerbation of the inflammatory response that subsequently diminishes after ALA-PDT. Additionally, posttreatment, there was a significant reduction in the volume of sebaceous glands. However, the involvement of the oxidized low-density lipoprotein receptor 1 (OLR1) and Wnt/β-catenin pathways has not been previously explored in the context of the effects of ALA-PDT on lipogenesis.

Our study revealed that OLR1, a class E scavenger receptor associated with atherosclerosis and involved in the activation of immune cells and inflammatory processes (Truthe et al. 2024; Sánchez-León et al. 2024), is a key mediator in the suppression of lipogenesis following ALA-PDT in both XL-i-20 sebocytes and an acne-like mouse model. Transcriptomic analysis revealed significant upregulation of OLR1 after treatment, which was associated with a marked reduction in lipid accumulation. Furthermore, our data indicated that ALA-PDT-induced activation of the Wnt/β-catenin pathway plays a crucial role in this process, with both Wnt/β-catenin inhibition and OLR1 knockdown leading to reversal of lipogenesis suppression. ROS play a pivotal role in the action of ALA-PDT, which was also observed post-treatment (Fig. 2C). It has been documented that ROS could activate the p38MAPK/NF-κB signaling pathway inducing OLR1 expression, and the administration of the ROS inhibitor NAC has been demonstrated to markedly attenuate ox-LDL-induced LOX-1 expression and cellular senescence (Zhao et al. 2014; Bing et al. 2024). Notably, an intricate interplay between LOX-1 and ROS may be involved in ox-LDL-induced mitochondrial DNA damage and autophagy (Ding et al. 2013). Consequently, the observed upregulation of OLR1 following ALA-PDT treatment may be intricately linked to the generation of ROS.

Mechanistically, the involvement of Wnt/β-catenin signaling, which plays a decisive role in regulating cellular fate and sebaceous gland homeostasis (Clayton et al. 2019), in acne pathophysiology has been proposed but remains underexplored. While in the liver, the formation of the β-catenin/mTOR complex could lead to the upregulation of lipid synthesis-related gene, promoting lipogenesis and resulting in hepatic steatosis (Wang et al. 2023). Liu A et al. revealed an inhibition of Wnt/β-catenin pathway in HNE-1 cells caused by EtNBSe-PDT, and perhaps the Wnt pathway represents another significant signaling cascade modulated by PDT, in addition to the mTOR pathway (Liu et al. 2019). Our study strengthens this hypothesis by showing that ALA-PDT-induced suppression of Wnt/β-catenin activity contributes to the downregulation of lipogenesis-related proteins such as SREBP1, FAS, and PPARγ. These findings were further corroborated by experiments in which Wnt/β-catenin activation reversed the therapeutic effects of ALA-PDT, suggesting a pivotal role for this pathway in regulating sebum production during PDT. Recently, ALA-PDT has been reported to induce mitochondrial stress and oxidative damage in SZ95 sebocytes (Jiang et al. 2024). The mitochondria-Wnt signaling axis has been identified, whereby a decrease in mitochondrial ATP could lead to impaired Wnt signaling and endoplasmic reticulum stress (Costa et al. 2019). Whether the suppression of the Wnt pathway subsequent to ALA-PDT is a consequence of mitochondrial ATP damage necessitates additional rigorous researches.

The clinical implications of our findings are significant. By identifying OLR1 as a critical receptor involved in the sebosuppressive effects of ALA-PDT, we propose that OLR1 could serve as a novel therapeutic target for enhancing the efficacy of photodynamic therapy in treating acne vulgaris. Furthermore, the Wnt/β-catenin pathway, known for its broad roles in regulating cellular differentiation and metabolism, may offer additional avenues for developing adjunctive therapies to optimize PDT outcomes.

While our findings provide valuable insights into the molecular mechanisms of ALA-PDT, some limitations must be acknowledged. First, the mouse model used in this study has inherent limitations in mimicking human acne, particularly with respect to sebum production on the skin surface. Second, the mechanism by which ALA-PDT upregulates OLR1 remains to be further elucidated. Future studies should focus on the regulatory networks that lead to OLR1 activation and its relationship with other key inflammatory mediators. Moreover, investigating the correlation between OLR1 expression levels and acne severity in clinical samples could offer important insights into its potential as a biomarker for ALA-PDT efficacy.

## Conclusion

In conclusion, this study provides compelling evidence that ALA-PDT upregulates OLR1 and activates the Wnt/β-catenin pathway to suppress lipogenesis in sebocytes. These findings open new avenues for the development of more effective acne treatments by targeting the molecular pathways identified herein. Future research will be crucial in translating these findings into improved therapeutic strategies for acne vulgaris.

## Data Availability

No datasets were generated or analysed during the current study.

## Abbreviations

ALA-PDT: 5-Aminolevulinic acid photodynamic therapy
ALA: 5-Aminolevulinic acid
PDT: Photodynamic therapy
OLR1: Oxidized low-density lipoprotein receptor 1
LOX-1: Lectin-type oxidized low-density lipoprotein receptor-1
SREBP1: Sterol regulatory element-binding protein-1
FAS: Fatty acid synthase
PPARγ: Peroxisome proliferator activated receptor gamma
ROS: Reactive oxygen species
AMPK: Adenosine 5‘-monophosphate-activated protein kinase
HBSS: Hank’s Balanced Salt Solution
DEGs: Differentially expressed genes
GO: Gene Ontology
LMRGs: Lipid metabolism related genes
DMSO: Dimethyl sulfoxide
CCK-8: Cell Counting Kit-8
RT‒PCR: Quantitative real-time PCR
IHC: Immunohistochemistry
H&E: Hematoxylin and eosin
WB: Western blot
JZ: Junction zone
NC: Negative control
