# Correction: Mechanistic insights into 5-aminolevulinic acid photodynamic therapy for acne vulgaris: targeting lipogenesis via the OLR1-Wnt/β-catenin pathway

**DOI:** 10.1186/s10020-026-01602-5

**Published:** 2026-08-14

**Authors:** Jia Yan, Linglin Zhang, Qingyu Zeng, Yitao Qian, Ke Li, Xiaojing Liu, Yun Wu, Yu Yan, Haiyan Zhang, Szeman Cheung, Jia Liu, Ronald Sroka, Xiuli Wang, Lei Shi

**Affiliations:** 1 Institute of Photomedicine, Shanghai Skin Disease Hospital, School of Medicine, Tongji University, Shanghai, 200092 China; 2 Laser-Forschungslabor, LIFE Center, University Hospital, Ludwig-Maximilian University, Planegg, 82152 Germany; 3 Department of Dermatology, Huadong Hospital, Fudan University, Shanghai, 200040 P. R. China

**Correction: Mol Med 31, 41 (2025)**

**https://doi.org/10.1186/s10020-025-01104-w**

In this article (Yan et al. 2025), Fig. 2 (D and F) appeared incorrectly and have now been corrected in the original publication. For completeness and transparency, the old incorrect versions are displayed below.

**Fig. 2:**
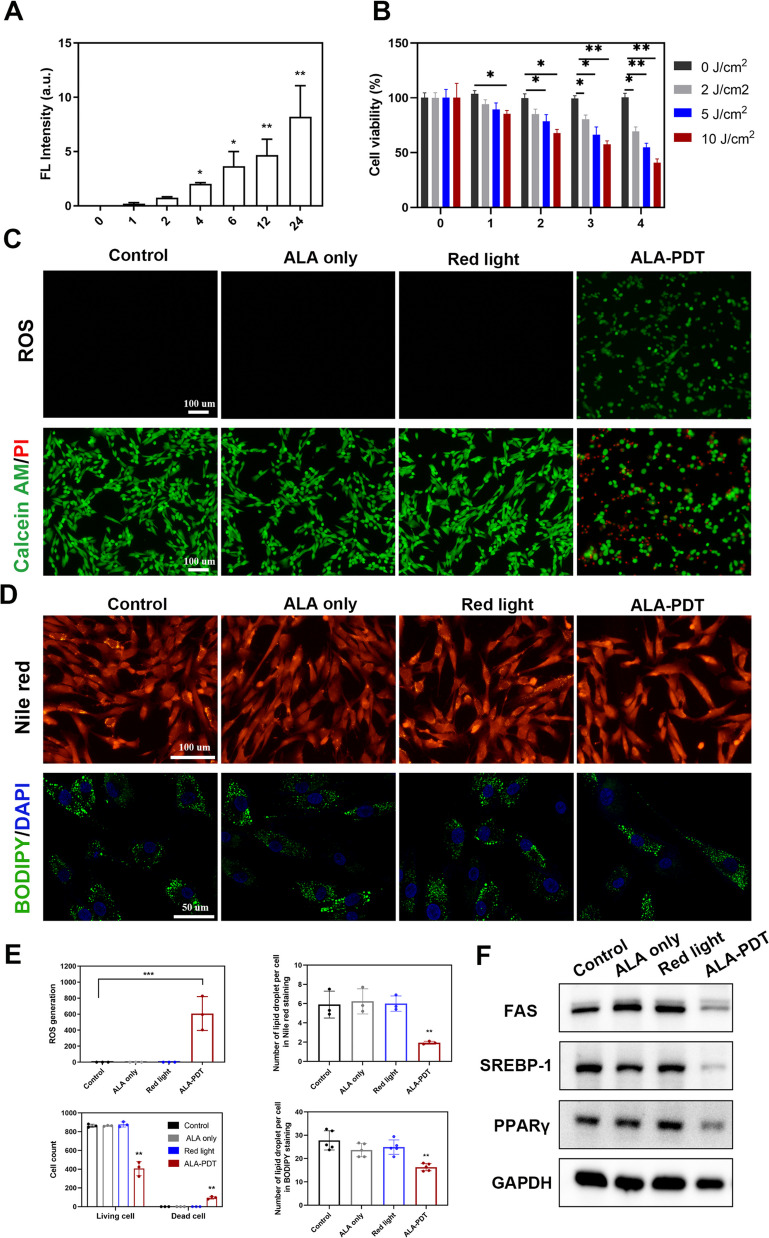
ALA-PDT inhibits lipid accumulation in XL-i-20 sebocytes. **A** Fluorescence intensity of XL-i-20 sebocytes after incubated with 0.5 mM ALA for 0–24 h detected via a fluorescence spectrophotometer. **B** Viability of XL-i-20 sebocytes incubated with 0.5 mM ALA for different durations and illuminated with red light at different doses. **C** ROS production and live/dead cell staining of XL-i-20 sebocytes were observed via fluorescence microscopy. **D** Nile red staining and BODIPY staining of XL-i-20 sebocytes 24 h after ALA-PDT. **E** ROS semiquantitative analysis and counting of living/dead cells in 2 C. Semiquantitative analysis of lipid droplets per cell in 2D. **F** Western blot of FAS, SREBP1 and PPARγ proteins in XL-i-20 sebocytes 24 h after ALA-PDT. *P < 0.05, **P < 0.01

**Fig. 2:**
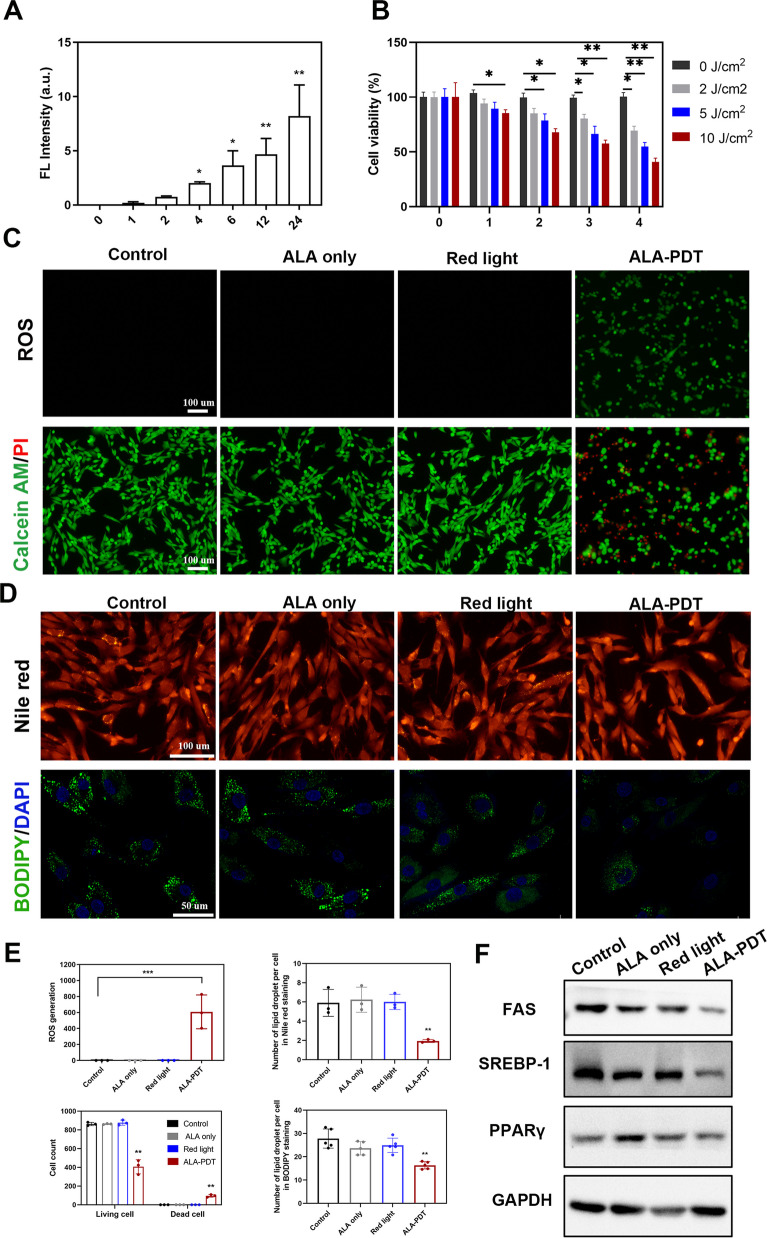
ALA-PDT inhibits lipid accumulation in XL-i-20 sebocytes. **A** Fluorescence intensity of XL-i-20 sebocytes after incubated with 0.5 mM ALA for 0–24 h detected via a fluorescence spectrophotometer. **B** Viability of XL-i-20 sebocytes incubated with 0.5 mM ALA for different durations and illuminated with red light at different doses. **C** ROS production and live/dead cell staining of XL-i-20 sebocytes were observed via fluorescence microscopy. **D** Nile red staining and BODIPY staining of XL-i-20 sebocytes 24 h after ALA-PDT. **E** ROS semiquantitative analysis and counting of living/dead cells in 2 C. Semiquantitative analysis of lipid droplets per cell in 2D. **F** Western blot of FAS, SREBP1 and PPARγ proteins in XL-i-20 sebocytes 24 h after ALA-PDT. *P < 0.05, **P < 0.01
